# Association between features of patient-provider discussions and routine prostate-specific antigen testing

**DOI:** 10.1371/journal.pone.0177687

**Published:** 2017-05-16

**Authors:** Joshua M. Liao, Mark J. Ommerborn, Cheryl R. Clark

**Affiliations:** 1 Division of General Internal Medicine, University of Pennsylvania School of Medicine, Philadelphia, PA, United States of America; 2 Center for Community Health and Health Equity, Brigham & Women’s Hospital, Boston, MA, United States of America; 3 Harvard Medical School, Boston, MA, United States of America; 4 Division of General Internal Medicine and Primary Care, Brigham and Women’s-Faulkner Hospitalist Program, Boston, MA, United States of America; Carolina Urologic Research Center, UNITED STATES

## Abstract

**Introduction:**

Although the US Preventive Services Task Force recommends against routine prostate cancer screening with prostate-specific antigen (PSA) testing, specialty organizations support screening via shared decision making between providers and selected patients. While discussions about advantages and disadvantages of testing are a feature of patient-centered care, it is unclear how provider recommendations and the presence of a personal doctor influence testing in the presence of such discussions.

**Materials and methods:**

We used the 2013 Behavioral Risk Factor Surveillance System to identify 1,737 male respondents surveyed about their PSA testing decisions. We describe the prevalence of provider recommendations and utilize weighted multivariable logistic regression models to examine the impact of provider recommendations and presence of a personal doctor on routine testing while accounting for patient-provider discussions about advantages and disadvantages.

**Results:**

The majority (70.4%) of respondents reported some form of discussion with providers about testing and most underwent screening in accordance with provider recommendations. In multivariable analyses, men whose providers had never recommended PSA test were less likely to receive screening [OR 0.03, 95% CI (0.02–0.05)], and patients who did not identify a personal doctor in their care were less likely to undergo testing [OR 0.12, 95% CI (0.04–0.32)].

**Discussion:**

Provider recommendations and having a personal doctor are associated with routine PSA testing. These findings suggest that providers and policymakers should be aware of how the content and context of communication with patients, beyond discussions of risks and benefits, can influence routine PSA testing behaviors.

## Introduction

The role of routine prostate cancer screening with prostate-specific antigen (PSA) testing continues to be debated [1]. Although overall testing rates have decreased in response to US Preventive Services Task Force recommendations discouraging routine screening [2,3], specialty organizations support screening via shared decision making between providers and selected patients [4,5].

Discussions about risks and benefits are a feature of patient-centered care [6] and can be associated with receipt of routine PSA screening [7]. In addition, testing decisions may also be affected by factors such as provider recommendations and continuity between patients and regular providers in their care. Understanding the impact of these factors on routine PSA testing in the presence of risk/benefit discussions can be important for increasing the quality of shared decision making. To date, however, such data have remained lacking.

In this analysis, we examine the association between these features of patient-provider discussions–provider recommendations about testing; continuity as defined by patients’ perception and ability to identify at least one “personal doctor” in their care–and the likelihood of routine PSA testing in men for whom shared decision making could be considered.

## Methods

### Study sample

We used the 2013 Behavioral Risk Factor Surveillance System, a national cross sectional tele- phone survey of non-institutionalized adults, to identify male respondents from the four states (Alabama, Delaware, Hawaii, Massachusetts) that surveyed men about their testing decisions.

We restricted our sample to respondents eligible for age-based prostate cancer screening via shared decision making using conservative sub-specialty guidelines^4,5^. This included male respondents aged 55 to 69 years who either reported receiving PSA screening as part of a routine exam or never receiving a PSA screening test. Respondents were excluded if they reported testing for any other reason, including personal or family history of prostate cancer, or if they were unsure why they underwent testing. Our analytic cohort included 1,737 men with complete data on PSA screening and covariates of interest.

### Statistical analysis

Descriptive statistics were calculated as weight-percentages. The odds of receiving routine PSA testing associated with provider recommendations and presence of a personal doctor were estimated with weighted logistic regression models adjusted for risk/benefit discussions and demographic, clinical, and access to care measures. Consistent with prior approaches^7^, we defined risk/benefit discussions via four patterns: discussions of advantages and disadvantages, only advantages, only disadvantages, and no discussion.

Statistical significance was determined with two-tailed Wald-F tests at the 0.05 alpha level. Analyses were performed in SAS-callable SUDAAN (version 9.0.1) using sampling weights to account for the complex survey design.

## Results

Overall, most of the men in our cohort were healthy, educated and able to adequately access health care (Table 1). The majority were white (80%) and in at least good health (83%), with fewer than 20% reporting diagnoses of cancer, cerebrovascular disease, diabetes or asthma. Over half had at last some college or technical school education (65%) and annual incomes over $50,000 (65%). The vast majority of our cohort also reported good health care access, with 96% being insured, 92% having a personal doctor in their care, and only 6.4% reporting delays in receiving care due to cost.

**Table 1:** Prevalence of PSA testing by covariates.

N = 1,737		Routine PSA test		
	Total	Yes	No	P value
**Patterns of Patient-Provider Discussions**				<0.001
No Discussion of Advantages or Disadvantages	583 (29.6)	87 (17.0)	496 (83.0)	
Discussion of Disadvantages	24 (1.5)	13 (36.6)	11 (63.4)	
Discussion of Advantages	610 (34.9)	522 (83.9)	88 (16.1)	
Discussion of Advantages and Disadvantages	520 (34.0)	440 (88.2)	80 (11.8)	
**Provider ever recommended PSA test**				<0.001
No	708 (37.4)	119 (18.5)	589 (81.5)	
Yes	1,029 (62.6)	943 (92.5)	86 (7.5)	
**Has a Personal Doctor**				<0.001
No	159 (7.7)	36 (18.9)	123 (81.1)	
Yes (one or more than one)	1,578 (92.3)	1,026 (68.6)	552 (31.4)	
**Age, Median (IQR)**	60 (57–64)	61 (58–65)	58 (56–63)	<0.001
**Race/ethnicity**				<0.001
Black	87 (5.7)	45 (57.5)	42 (42.5)	
Asian/Pacific Islander	226 (10.9)	98 (40.1)	128 (59.9)	
Hispanic	75 (3.7)	38 (61.8)	37 (38.2)	
White	1,349 (79.7)	881 (68.9)	468 (31.1)	
**Education**				<0.001
Less than High School	68 (6.9)	35 (58.2)	33 (41.8)	
High School	403 (27.9)	199 (51.4)	204 (48.6)	
Some College / Technical School	414 (25.4)	223 (62.5)	191 (37.5)	
College / Technical School or more	852 (39.8)	605 (76.9)	247 (23.1)	
**Income**				<0.001
< $25,000	388 (15.8)	159 (46.7)	229 (53.3)	
$25,000 to < $50,000	373 (19.4)	215 (59.1)	158 (40.9)	
$50,000 to < $75,000	281 (19.0)	188 (71.8)	93 (28.3)	
≥ $75,000	695 (45.8)	500 (70.6)	195 (29.4)	
**Self-rated health**				<0.05
Fair or Poor	312 (16.8)	154 (53.8)	158 (46.2)	
Good	513 (30.2)	296 (61.7)	217 (38.4)	
Very Good	581 (34.0)	384 (71.1)	197 (28.9)	
Excellent	331 (19.0)	228 (68.4)	103 (31.6)	
**Cancer Diagnosis**				<0.001
Yes	298 (16.9)	216 (78.7)	82 (21.3)	
No	1,439 (83.1)	846 (62.0)	593 (38.0)	
**Diabetes**				0.54
Yes	275 (15.5)	156 (62.1)	119 (37.9)	
No	1,462 (84.5)	906 (65.3)	556 (34.7)	
**Asthma**^**a**^				0.39
Yes	184 (11.4)	124 (70.5)	60 (29.5)	
No	1,553 (88.6)	938 (64.1)	615 (35.9)	
**Cerebrovascular Disease**				0.87
Yes	243 (13.0)	141 (64.1)	102 (35.9)	
No	1494 (87.0)	921 (64.9)	573 (35.1)	
^**b**^**Insurance Status**				<0.001
No	90 (4.2)	20 (16.0)	70 (84.0)	
Yes	1,647 (95.8)	1,042 (67.0)	605 (33.1)	
**Delayed Care due to cost**				<0.01
Yes	121 (6.4)	49 (42.8)	72 (57.3)	
No	1,616 (93.7)	1,013 (66.3)	603 (33.7)	

**Source**. Data from Centers for Disease Control and Prevention 2013 Behavioral Risk Factor Surveillance Survey. Analysis performed among the N = 1,737 participants with complete data on all covariates. **Notes**.

^a^Yes, includes present or former asthma status.

^b^Insurance status is a point estimate and does not indicate whether the respondent was continually insured.

The majority (70.4%) of respondents reported some form of discussion with providers about testing, and most (62.6%) reported that their providers recommended screening (Table 1). Patients tended to undergo PSA screening in accordance with provider recommendations (92.5% underwent screening when recommended; 81.5% did not when it was not recommended). Men who had no discussions with providers were unlikely to receive screening (83.0%), while those who discussed advantages and disadvantages were likely to screen (88.2%).

In multivariable analyses, men whose providers had never recommended a PSA test were less likely to receive screening [OR 0.03, 95% CI (0.02–0.05)] than those whose providers had ever recommended it (Table 2). Similarly, patients who did not identify a personal doctor in their care were less likely to undergo testing compared to those who did [OR 0.12, 95% CI (0.04–0.32)].

**Table 2:** Odds ratios for PSA testing as part of routine care in the United States.

Odds Ratios^a^ (95% CI)		
N = 1,737	Unadjusted	Fully Adjusted^b^
**Provider ever recommended PSA test**		
No		0.03 (0.02–0.05)
Yes		1.00
**Has a Personal Doctor**		
No		0.12 (0.04–0.32)
Yes (one or more than one)		1.00
**Patterns of Patient-Provider Discussions**		
No Discussion of Advantages or Disadvantages	1.00	1.00
Discussion of Disadvantages	2.82 (0.80–9.91)	0.59 (0.15–2.31)
Discussion of Advantages	25.46 (14.46–44.81)	5.35 (2.76–10.38)
Discussion of Advantages and Disadvantages	36.50 (21.33–62.47)	6.04 (3.13–11.64)
**Age**		1.15 (1.08–1.23)
**Race**		
Black		1.00 (0.36–2.73)
Asian/Pacific Islander		0.29 (0.16–0.52)
Hispanic		1.16 (0.39–3.48)
White		1.00
**Education**		
Less than High School		2.86 (0.78–10.49)
High School		0.38 (0.17–0.86)
Some College / Technical School		0.86 (0.43–1.72)
College / Technical School or more		1.00
**Income**		
< $25K		0.49 (0.21–1.13)
$25K to < $50K		0.87 (0.40–1.91)
$50K to < $75K		1.10 (0.42–2.91)
≥ $75K		1.00
**Self-rated health**		
Fair or Poor		0.50 (0.15–1.71)
Good		1.12 (0.46–2.73)
Very Good		1.22 (0.56–2.65)
Excellent		1.00
**Cancer Diagnosis**		
Yes		1.61 (0.66–3.91)
No		1.00
**Diabetes**		
Yes		0.87 (0.43–1.77)
No		1.00
**Asthma**^**c**^		
Yes		1.94 (0.97–3.89)
No		1.00
**Cerebrovascular Disease**		
Yes		1.19 (0.58–2.44)
No		1.00
^**d**^**Insurance Status**		
No		0.45 (0.16–1.29)
Yes		1.00
**Delayed Care due to cost**		
Yes		0.83 (0.37–1.85)
No		1.00

**Source**. Data from Centers for Disease Control and Prevention 2013 Behavioral Risk Factor Surveillance Survey. Analysis performed among the N = 1,737 participants with complete data on all covariates. **Notes**.

^a^Multivariable logistic regression models weighted with rlogist function in SUDAAN.

^b^Model adjusted for age, race/ethnicity education, income, self-rated health, cancer diagnosis, diabetes, asthma, cerebrovascular disease, insurance status, and delayed care due to cost.

^c^Yes, includes present or former asthma status.

^d^Insurance status is a point estimate and does not indicate whether the respondent was continually insured.

Compared to men who had no discussions about advantages or disadvantages of PSA testing with providers, those who discussed only advantages [OR 5.35, 95% CI (2.76–10.38)], or dis- cussed both [OR 6.04, 95% CI (3.13–11.64)] were more likely to undergo testing. No significant differences were observed for men who only discussed disadvantages with providers [OR 0.59, 95% CI (0.15–2.31)] compared to those who had no discussions. Results were robust to adjustment for patient age, race/ethnicity, education, income, self-rated health, co-morbid conditions, insurance status, and delayed care due to cost.

## Discussion

Our results underscore that routine PSA testing is not only associated with discussions of risks and benefits [7], but also provider recommendations about testing and patients’ perceived continuity or familiarity with providers in their care. These findings are notable for several reasons.

First, although shared decision making involves eliciting patient preferences and values, providers also impart their values through their recommendations, as well as their framing of risks and benefits. Our analysis builds on earlier work done before broad adoption of shared decision making [8] and is the first to demonstrate that–in the presence of widespread discussions about risks and benefits–provider recommendation remains a strong driver of routine testing.

This reflects the fact that recommendations about PSA testing and discussions about PSA testing are related, but distinct, issues. Additional research should therefore seek to understand how provider motivations and values drive recommendations, as well as test the independent and combined effects of recommendations and discussions on PSA testing behavior. Ultimately, a combination of patient- and provider-focused policies that emphasize both appropriate recommendations as well as balanced discussions of risks and benefits are needed to improve the appropriateness of routine PSA testing.

Second, the association between presence of a personal doctor and routine PSA testing also suggests that patients’ perceived continuity or familiarity with providers can influence testing independent of provider recommendations or discussions of risks and benefits. One implication of this result is that future work should evaluate how PSA testing behaviors are influenced by the context (where and with whom) as well as the content (discussion of advantages and disadvantages; provider recommendations) of shared decision making.

Our analysis is limited by an inability to determine causality, as well as potential underreporting and sampling, recall or non-response bias in a non-nationally representative sample. The sample also included small numbers of Hispanic or African American men (9.4% of the cohort), potentially high risk groups for whom alternative screening approaches may be indicated.

Nonetheless, our findings suggest that providers and policymakers should be aware of how the content and context of communication with patients, beyond discussions of risks and benefits, can influence routine PSA testing behaviors.
